# “We must help them despite who they are…”: healthcare providers’ attitudes and perspectives on care for young gay, bisexual and other men who have sex with men in Nairobi, Kenya

**DOI:** 10.1186/s12913-023-10026-4

**Published:** 2023-10-03

**Authors:** Samuel Waweru Mwaniki, Peter Mwenda Kaberia, Peter Mwangi Mugo, Thesla Palanee-Phillips

**Affiliations:** 1 School of Clinical Medicine, Faculty of Health Sciences, University of the Witwatersrand, Johannesburg, South Africa; 2 Department of Health Services, Administration and Campus Support Services, University of Nairobi, Nairobi, Kenya; 3 Department of Mathematics, Faculty of Science and Technology, University of Nairobi, Nairobi, Kenya; 4 Kenya Medical Research Institute - Wellcome Trust Research Programme, Nairobi, Kenya; 5 Wits Reproductive Health and HIV Institute, Faculty of Health Sciences, University of the Witwatersrand, Johannesburg, South Africa; 6 Department of Epidemiology, School of Public Health, University of Washington, Seattle, WA USA

**Keywords:** Challenges, Healthcare workers, Key populations, Opportunities, Sensitization, Training, Young men who have sex with men (YMSM)

## Abstract

**Background:**

Compared to young heterosexual men, young gay, bisexual and other men who have sex with men (YMSM) face a disproportionate burden of sexual health conditions. This disparity is occasioned by factors such as criminalization and stigmatization of same-sex practices, YMSM’s limited access to non-judgmental and non-discriminatory health services, and challenges associated with healthcare delivery. We explored the attitudes and perspectives of tertiary academic institution-based healthcare providers (HCPs) toward provision of services to YMSM in Nairobi, Kenya.

**Methods:**

In September 2021, six in-person focus group discussions (FGDs) were held with 36 HCPs drawn from six public tertiary academic institutions within the Nairobi metropolis. HCPs were drawn from six cadres: front office staff, nurses, clinicians, counsellors, laboratory technologists, and pharmaceutical technologists. Discussions were conducted in English, transcribed verbatim and analyzed thematically using NVivo version 12.

**Results:**

Analysis showed that despite expressing disapproval of same-sex practices, HCPs recognized their professional duty to provide care to YMSM, voiced challenges they experienced when providing care to YMSM, and suggested possible strategies for improving care for YMSM. Disapproval of same-sex practices mainly stemmed from HCPs’ personal values, societal norms and religious beliefs, though some HCPs identified religious principles such as the golden rule of “treating others as one would want to be treated” as motivation to providing care to YMSM. HCPs did not perceive criminalization of same-sex practices as a barrier to providing care to YMSM. Healthcare delivery challenges included inadequate knowledge and skills, a desire to “convert” YMSM’s perceived deviant homosexual to the normative heterosexual orientation, secondary stigma from other HCPs, and healthcare settings that did not support YMSM to disclose same-sex practices. Suggestions for improving care comprised sensitization and training of HCPs, encouraging more HCP-YMSM interaction, providing YMSM-friendly and inclusive services, and advocacy for YMSM services.

**Conclusion:**

There is need for interventions to improve HCPs’ knowledge of YMSM’s health needs, build skills to respond to these needs, and foster affirming attitudes toward same-sex practices. By so doing, YMSM can hopefully be able to access services that meet their needs, and are non-discriminatory, non-stigmatizing and non-judgmental.

**Supplementary Information:**

The online version contains supplementary material available at 10.1186/s12913-023-10026-4.

## Introduction

During the developmental stage of emerging adulthood (18–25 years), individuals establish their identities, develop values, form new relationships with peers and test new levels of independence [1]. It is also during this period that sexual exploration and experimentation which may have begun in adolescence more likely intensifies, and may involve risky behaviours such as sexual interactions outside committed relationships [1]. This may in turn result in harmful effects on emerging adults’ sexual health. Meyer’s Minority Stress Model explains how discrimination, prejudice and stigma create a hostile social environment that results in health inequities among sexual and gender minority populations [2]. Based on this model, young gay, bisexual and other men who have sex with men (referred to here as YMSM) may experience particular social stressors including low levels of parental support [3], school-based victimization [4] and gender role strain [5] – factors which in combination with the vulnerabilities of the emerging adulthood developmental phase, may act synergistically to compromise their health and well-being [6]. For instance, there is evidence to suggest YMSM are at increased risk of human immuno-deficiency virus (HIV) and other sexually transmitted infections (STIs) such as chlamydia and gonorrhea [7].

At the cusp of emerging adulthood (age of 18 years), many students transition from secondary to tertiary educational institutions, where they find themselves in an environment with decreased direct supervision of their behaviour and increased freedom [8]. For tertiary student YMSM this may involve a sudden exposure to peers with similar sexual orientation/behaviour, and more opportunities and freedom to socialize. These new interactions may indeed confer psychosocial benefits such as creating a sense of belonging, and promoting the processes of coming out and identity development for YMSM [9]. However, the freedom and decreased direct supervision of behaviour, may lead to YMSM engaging in risky sexual behaviours such as condomless anal sex, sex work, group sex and sexualized drug use [10], hence increasing their risk for HIV/STI infections. Other structural factors such as criminalization of male same-sex practices [11], arbitrary arrests and convictions of YMSM [12], homophobia [13] as well as violence against MSM [14], work in concert to further negatively impact the health and well-being of YMSM, including those in tertiary academic institutions.

Moreover, the plethora of health challenges experienced by YMSM are amplified by inadequate access to appropriate health services that address the unique health needs of YMSM [15]. Barriers to service access include: hostile healthcare settings that are unconducive for YMSM to disclose and openly discuss same-sex practices with healthcare providers (HCPs) [16], lack of provider knowledge on the unique health needs of YMSM [17], as well as experiences of stigma and discrimination in healthcare settings [18]. Discussing same-sex practices with HCPs is important as this may facilitate appropriate healthcare engagement, including risk assessment for HIV/STI infections [19, 20]. However, studies carried out in various countries within sub-Saharan Africa show that once HCPs learn of clients’ same-sex practices, then such clients including YMSM experience sexual stigma and discrimination from the HCPs, and this may act as a barrier to access to appropriate health services [21–26]. Additionally, HCPs in Africa receive limited or no training in sensitive management of the sexual health needs of YMSM, further impacting negatively on the scope and efficacy of HIV/STI control measures among YMSM [27]. Within the African continent, debates in the general public about the legality and morality of same-sex practices focus deleterious attention on YMSM, thereby compounding the aforesaid barriers to care seeking by YMSM and service provision by HCPs [28].

In Kenya, same-sex practices are criminalized [29]. However, in a February 2023 ruling, Kenya’s Supreme Court upheld the decision of lower courts that ordered the non-governmental organizations coordination board to register a human rights organization (National Gay and Lesbian Human Rights Commission) which the board had declined to register because it had the words ‘gay’ and ‘lesbian’ in its name [30]. In terms of healthcare, there is a dedicated national program within the ministry of health that leads the HIV/STI response among key populations, including MSM [31]. MSM access services mainly from MSM-friendly clinics which are run by community-based or non-governmental organizations [32]. Kenyan MSM have previously reported experiences of stigma and discrimination in the hands of HCPs when seeking sexual health services from public health facilities [33]. Given there is only a handful of MSM-friendly clinics in the country, it is important to understand the attitudes and perspectives of HCPs toward care for MSM in other healthcare settings where MSM are likely to seek services.

Some of the aforementioned health challenges were observed in our recent HIV/STI bio-behavioural survey among YMSM in Nairobi, Kenya [34, 35]. Based on this survey, HIV prevalence among YMSM was estimated to be 3.6% which is six times higher than that of young Kenyan heterosexual men aged 18–24 years [36]. We also observed pervasive prevalence of STI, with more than half (58.8%) of study participants testing positive for at least one of five curable STIs (chlamydia, *Mycoplasma genitalium* infection, gonorrhea, trichomoniasis or syphilis) [37]. During qualitative in-depth interviews, YMSM relayed experiences of prejudice, stigma and discrimination from HCPs when seeking care in public and tertiary institution-based health facilities, but reported being fairly treated in community pharmacies, private and YMSM-friendly health facilities [38]. In light of these earlier outcomes, there is a critical need to better understand the attitudes and perspectives of HCPs toward provision of care to YMSM, so as to inform development of interventions aimed at improving services for YMSM. In the current study, we sought to assess the attitudes and perspectives of tertiary institution-based HCPs toward provision of services to YMSM, in Nairobi, Kenya.

## Methods

The study methods are summarized in the published study protocol [34], and detailed below:

### Study design and approach

This was a qualitative study in which data were collected through focus group discussions (FGDs) with HCPs. FGDs were selected for their demonstrated usefulness in exploratory research in under-researched topics [39], and their effectiveness in helping participants to constructively speak about a research topic that is usually difficult to talk about [40].

### Setting

The study was conducted in Nairobi, the capital city of Kenya, where the preceding studies that estimated the prevalence of HIV/STI infections, and investigated healthcare engagement among YMSM were conducted [36–38]. The capital city and its metropolis is home to approximately 150 campuses of various universities and mid-level colleges [41], some of which have health facilities that serve the student population [42–44].

### Participants, sampling and recruitment

HCPs were eligible if they directly provided services to students in a health facility based in a tertiary institution within the Nairobi metropolis, and provided informed consent to participate in the study. HCPs were drawn from the following cadres of staff: front office staff (who are usually health records personnel), nurses, clinicians, counsellors, pharmaceutical and laboratory technologists. HCPs were purposively recruited (based on their professional cadres) by the heads of their health facilities. HCPs were recruited from six public universities that have health facilities which provide services to the student population. One HCP per cadre per site was invited to participate, though some facilities were allowed to nominate two participants from one cadre in cases where they did not have a HCP in another cadre to attend. Thirty-six [36] HCPs took part in the study.

### Data collection procedures

Demographic information was collected through a short paper-based survey. The FGD guide was developed by the researchers in line with the aim of the study (supplementary file 1). The guide covered two main topics, namely: attitudes (subjective feelings or emotions) and perspectives (both objective and subjective perceptions) of HCPs toward providing care to YMSM. Examples of questions under the topic of attitudes were: how would you feel if you came to know a student seeking health services from you was MSM, and would you say you have any feelings/beliefs/attitudes/opinions that would make it difficult for you to provide services to MSM students? For perspectives, examples of questions included: would you say that MSM students are able to access sexual health services from the health facilities in the learning institutions, and have you had experience providing services to MSM students? The FGD sessions were held in the month of September 2021, at a private conference centre within the Nairobi central business district. Sessions were facilitated by the first and second authors, who were each assisted by a co-facilitator who took notes and highlighted areas where the facilitators needed to prompt participants further to obtain deeper and clearer understanding of the discussion topics. A FGD consisted of HCPs from one cadre to ensure homogeneity, minimize the effects of group power dynamics and encourage more openness during discussions. One FGD was held for every cadre, resulting in six FGDs in total. Six FGDs were deemed adequate since previous research has shown that the most prevalent themes within a dataset can be identified with up to three FGDs [45]. Each FGD had five to seven participants, lasted an average of 2 h, and was conducted in English which is the language of instruction in the Kenyan education system. At the end of every discussion, the facilitators and co-facilitators held a 15–20 min debrief session to discuss preliminary findings and identify opportunities for improving subsequent sessions. All FGDs were audio-recorded and transcribed verbatim. The facilitators then read the transcripts while listening to the audio-recordings to ensure that the transcripts were accurate.

### Data analysis and reporting

Data were managed using NVivo software version 12 (QSR International). Analysis followed the content analysis theoretical framework which systematically organizes qualitative data in a structured manner [46]. Analysis adopted both deductive and inductive approaches where themes set a priori and emerging ones were coded respectively. Coding was done independently by two members of the study team, who then compared codes for agreement until a consensus was reached on whether to merge some codes, get rid of others or come up with new ones. Themes and sub-themes were described and supported with illustrative excerpts from the discussions. Excerpts were attributed to participants using their cadres and self-identified gender. Reporting of the study followed the consolidated criteria for reporting qualitative studies (COREQ) [47].

### Data trustworthiness

Four criteria (credibility, dependability, transferability and confirmability) were used to ensure trustworthiness of the data [48]. For credibility, the study used well-grounded methods for qualitative investigation. Prior to data collection, the first author held meetings with the heads of the tertiary institution-based health facilities to familiarize himself with how the health facilities conducted their operations. During these meetings, the heads of the health facilities were also briefed on the findings of the prior HIV/STI bio-behavioural survey, and qualitative investigation on healthcare engagement among YMSM [36–38]. This helped in obtaining a buy-in from these leaders, whose support was enlisted in recruiting HCPs from their respective facilities to the study. Member checking was done “on the spot” by paraphrasing and summarizing what the participants said at intervals and at the end of each topic of discussion, respectively. This helped to confirm meaning and facilitate deeper understanding of the information provided by HCPs. To address dependability, the study processes have been reported in sufficient detail to enable future researchers to reproduce the study, even if not necessarily to obtain similar results. For transferability, details about the study methods have been provided to help comprehend and compare/contrast the findings of this study with those of similar studies. Confirmability has been addressed by supporting the findings with verbatim quotes from study participants.

### Ethical considerations

The study protocol was approved by University of the Witwatersrand Human Research Ethics Committee-Medical (Reference number M200215) and University of Nairobi – Kenyatta National Hospital Ethics and Research Committee (Reference number P990/12/2019). Participants provided written informed consent before taking part in the study. During the discussions, participants were provided with an A4 white paper on which they wrote an allocated number by which they referred to each other as the discussions went on, in order to conceal one another’s identity. Participants were reassured to feel free to engage, and that there was no right or wrong answer. They were asked to keep what they heard during the discussions to themselves and not to divulge this outside the group. Participants were offered refreshments, and received Kenyan shillings 1,000 (approximately $10 at the time) each for travel-related expenses to and from the study site.

## Results

### Characteristics of study participants

The sociodemographic characteristics of study participants are shown in Table 1. A majority of the participants were female (63.9%), more than half (52.7%) were between the ages of 25–40 years, and most identified as Christians (94.4%). Distribution across cadres was almost similar with each cadre having 5–7 participants. More than half (55.5%) had a bachelor’s degree or higher. More than half (52.8%) had a work experience of 11–20 years since first qualification from college/university, with a similar proportion having worked in a tertiary institution-based health facility for 6–10 years, indicating that a significant proportion had worked in a place other than a tertiary institution-based health facility.

**Table 1:** Sociodemographic characteristics of healthcare providers participating in FGDs (N = 36)

Variable	Category	n (%)
Self-identified gender	Woman	23 (63.9)
	Man	13 (36.1)
Age in years	25–40	19 (52.7)
	41–50	17 (47.3)
Religion	Christianity	34 (94.4)
	Islam	2 (5.6)
Cadre	Clinician	7 (19.4)
	Nurse	7 (19.4)
	Counsellor	6 (16.7)
	Front office staff	6 (16.7)
	Laboratory technologist	5 (13.9)
	Pharmaceutical technologist	5 (13.9)
Highest level of education attained	Diploma	12 (33.3)
	Higher national diploma	4 (11.1)
	Bachelor’s degree	17 (47.2)
	Master’s degree	3 (8.3)
Work experience in years since first qualification from college/university	1–10	9 (25.0)
	11–20	19 (52.8)
	Above 20	8 (22.2)
Work experience in years in a tertiary institution-based health facility	1–5	8 (22.2)
	6–10	19 (52.8)
	11–20	9 (25.0)

FGDs: focus group discussions

The following section describes the three themes that emerged from data analysis.

### HCPs were obligated to provide care to YMSM despite having non-affirming attitudes toward same-sex practices

A majority of HCPs noted that they were professionally trained and had a duty to offer services to, and treat all clients equally, regardless of the clients’ sexual orientation and/or behaviour. As such, they perceived themselves as having a positive attitude toward providing care to YMSM.

“As health workers, we have to accept these people (YMSM) as our clients and treat them the way we treat other clients and give them help as we ought to, because when we were trained, we were not told that we should only treat those who we think are straight. So I think our attitude is positive because these are people who have come for help and we must help them despite who they are.” – Clinician, woman.

Other HCPs noted that they had no beliefs or opinions that would hinder them from offering services to YMSM, as long as they had the requisite resources to offer the services.

“Personally, I think there is no belief or opinion that will hinder me from offering services to such a person (YMSM) because I don’t see the reason why they shouldn’t be able to get services. I wouldn’t be uncomfortable in any way, so long as I have everything that I need to help such a person, I will do it.” – Laboratory technologist, woman.

However, some HCPs observed that homosexuality was against their personal values, as well as cultural and religious beliefs. Nevertheless, the HCPs stated that they were able to compartmentalize their values and beliefs on one side, and their professional obligation to offer services on the other side. In doing so, the HCPs believed they were able to offer services to YMSM, and treat them with dignity.

“Professionally, I would serve this patient the way I would serve any other patient. Ethically, how we have been brought up and according to my own judgement, whatever this person is doing (having sex with other men) is not right. Still, I would treat this patient with the dignity they deserve.” – Front office staff, man.

“My Christianity background does not allow me to accept that (homosexuality) as a way of life… I believe even Muslims don’t accept it. But I have agreed that I’ll bend backwards to accommodate the other person because of my profession… to do the best that I can to help where I can.” **–** Pharmaceutical technologist, woman.

On the other hand, some HCPs noted that it was the application of religious values such as the ‘golden rule’ (treating others the way one would like to be treated), and ‘accepting those rejected by society’, that had helped them come to a place where they were comfortable providing services to YMSM without judging.

“I have come to accept everybody the way they are … and to do unto others what I would like to be done unto me. So I just assist where I can and without judgment.” – Clinician, woman.

“I am guided by Christian principles because the person who I follow, the Christ, was a person who basically came here for those people who were considered the scum of the earth. So, I act in a very professional manner without prejudice, and give the service that is due to them (YMSM).” – Nurse, man.

Nevertheless, some HCPs who strongly held to their beliefs emphasized the importance of keeping those beliefs, but acknowledged the need to change judgmental attitudes toward YMSM, and seeking to understand YMSM and their needs, so as to facilitate better service provision.

“Changing what I believe because of someone’s behaviour (same-sex practices) is a no. I think what needs to be changed is the attitude… we need to be open minded and instead of judging them (YMSM), seek to understand them so we can help them better.” – Front office staff, woman.

Of note, while HCPs were aware of the criminalization of same-sex practices in the Kenyan law, this did not appear to be a barrier to offering services to YMSM. HCPs were clear of their responsibility to provide services to all in an equitable manner, drawing on the analogy of how they serve other clients who are alleged to engage in illegal activities such as stealing, without prejudice. They also noted that they had no role to play in addressing the criminalization of same-sex practices, but were instead focused on handling the health issues clients presented with at the time.

“If a student came to you after getting beaten for allegedly stealing, are you going to apply the same principle and say stealing is illegal and so I am not going to treat you? Or does it just apply to MSM? No. You have to treat everyone equally. Ours is to offer the services as we are required to do. The criminalization of homosexuality is really none of our business.” – Front office staff, man.

### HCPs encountered challenges when providing services to YMSM

It emerged that HCPs faced various challenges when providing services to YMSM. Five major types of challenges were described:

#### Inadequate knowledge and skills to serve YMSM

Despite acknowledging their duty of care to YMSM, HCPs noted that they felt limited by lack of the requisite knowledge and skills, for instance in the occurrence and management of anal STIs among YMSM. They attributed this to the little pre-service training that is offered on addressing health issues faced by sexual minority populations.

“I have always felt I am incapable in terms of knowledge and skills… I don’t feel I have the capacity to integrate MSM services into my daily work. I find I am incapable because the knowledge that is given to us in college is very minimal and so sketchy. For instance, I never heard about anal STIs in college.” **–** Nurse, female.

#### Parental/provider role conflict when attending to YMSM

Holding the perception that same-sex practices were deviant, some HCPs felt conflicted between their roles as parents and HCPs. They assumed a parental role over YMSM and felt the need to ask them to stop same-sex practices, and instead convert to the socially accepted heterosexual orientation. However, on the recognition of their professional obligation to provide care, HCPs stated that they were able to separate their parental and HCP roles, and thus provide services to YMSM in a non-judgmental manner.

“The first time I met these guys (YMSM), I was left wondering, “what if this was my son? Will I encourage him to continue with whatever he is doing (having sex with men), or ask him to stop?“ It was a challenge to me and I felt bad. But later on, I realized I am a counsellor and from there on I learned how to walk with (support) them without judging.” – Counsellor, woman.

#### Focusing on converting YMSM to heterosexual at the expense of attending to their health needs

Once they learnt of a client’s same-sex practices, some HCPs desired and attempted to ‘convert’ YMSM clients to being heterosexual. The HCPs did this at the expense of addressing the health needs, which may not have been necessarily related to the client’s same-sex practices. In the process, HCPs were likely to be overly inquisitive about the sexual behaviour of YMSM, asking sensitive questions to satisfy their curiosity, instead of questions whose responses to would help HCPs make decisions to address the health needs of the clients.

“But what I have realized, in the counselling centre, mostly, the first thing we do with MSM is trying to convert them (to heterosexual). We try to make them feel that this behaviour (same-sex practices) is not right, even if they have come to seek help for unrelated issues. Maybe they just have general counseling needs like any other client, but you find that we want to know, what is this? How did you become? When did you start? We start asking these type of questions for our own benefit, not for their needs.” – Counsellor, woman.

#### Experiences of secondary stigma from other HCPs for assisting YMSM clients

HCPs who were friendly to YMSM noted that the YMSM they provided services to referred their peers who needed services to the friendly HCPs. YMSM-friendly HCPs were concerned about judgmental attitudes from their colleagues who noticed that these HCPs were constantly sought out by clients who the colleagues perceived to be YMSM. To avoid this judgmental attitudes, the YMSM-friendly HCPs were occasionally forced to attend to YMSM at health facilities outside the institutions.

“You fear. How will the other staff see me if I am interacting with these people (YMSM) so much? Will they misjudge me and say I am also gay? So that hinders us sometimes from caring for these students and sometimes we might give them an appointment somewhere else outside the university clinic.” – Nurse, man.

#### Unconducive environments for YMSM to disclose and discuss same-sex practices with HCPs

Due to the non-acceptance of YMSM in society, HCPs noted that it was difficult for YMSM to seek care. When they did, YMSM were unwilling to disclose and discuss same-sex practices with HCPs. This, the HCPs felt limited their ability to offer optimum care to YMSM.

“Because MSM are not accepted in the society, they find it very hard to seek medical attention and even if they do, they are not willing to come out clean (disclose and discuss same-sex practices) so that you can help them appropriately.” **–** Clinician, woman.

### HCPs proposed strategies for improving health services for YMSM

Participants suggested various strategies that could be deployed to improve access to and use of health services by YMSM. Analysis revealed five strategies targeted at HCPs, facilities, YMSM and the public.

#### Sensitizing and training HCPs on healthcare needs of YMSM

The need to sensitize HCPs on how to handle YMSM within the academic institutions was highlighted. Such sensitization would help HCPs treat YMSM in a non-prejudicial and non-stigmatizing manner, so that YMSM would feel free to seek services.

“We need to sensitize staff that some of our students are MSM and we need to embrace them… when they come to your office don’t judge them, just serve them the way they come… because they are really stigmatized. If we continue to judge and stigmatize them, then they might not want to come to seek help.” – Clinician, man.

In addition, HCPs pointed out that they needed to be trained on the health needs of YMSM and how to treat them when they come to seek care.

“We need to be trained on MSM, how to treat them based on their health needs so that when they come to the clinic, even the health worker who is going to attend to them knows what to do.” – Nurse, woman.

#### Encouraging more HCP-YMSM interactions

HCPs felt that more interaction with YMSM in the line of duty would be helpful in changing their attitudes toward YMSM, and fostering an understanding that YMSM have a right to be who they are and access health services.

“I think time can make us change our opinions and attitudes. Because, as time goes by, you encounter more MSM than the ones you have encountered before, and also get to understand that it is their human right to have whatever sexual orientation they have and still get services.” **–** Laboratory technologist, woman.

#### Reaching out to YMSM

HCPs observed that it was paramount to reach out to prospective and continuing students with messages that YMSM were welcome to seek health services within the institutions. The orientation program for first year students was underscored as an apt opportunity to pass this information.

“During our orientation programs, we usually share about various issues with the students who are coming in because they are coming into a place where there is freedom and they have these kind of things happening… MSM, lesbianism and all that… So I think one of the things we can improve is to tell them that in our clinics we do not discriminate against them, so that they don’t miss an opportunity for healthcare because they’re afraid of how they will be perceived if they come to seek help.” **–** Nurse, man.

The other outreach strategy that was suggested was the use of social media to deliver creative messages inviting YMSM to seek services. This was thought to be an appropriate avenue since students in general are ardent users of social media.

“We should come out creatively through the social media. It is the easiest way because they actually identify with social media and use it a lot. We can be sending some attractive messages to encourage them (YMSM) to come out and access services.” – Counsellor, woman.

#### Offering YMSM-friendly health services

Participants conceptualized YMSM-friendly health services as comprising three components: having friendly HCPs, offering integrated services, and creating an inclusive climate. HCPs noted that when YMSM were treated well at the clinic, they referred their partners and friends to visit the health facilities and look out for the YMSM-friendly HCPs.

“By being friendly to them (YMSM) and serving them well, they realize they are not rejected. They feel “yes, I can come if I need help.” In turn, they normally tell their friends and partners that, “if you go, ask for so and so” because they know that they will get friendly services from this person.” **–** Counsellor, woman.

One HCP gave an example of how their friendliness resulted in many YMSM being referred to them for treatment of anal warts, even from other colleges and universities.

“I once treated one who had perianal warts. I knew it was an STI so I didn’t bother to ask him many questions about how he got the warts. I just treated him and followed up with him until he recovered fully. After that, they started coming in so many even from other universities. I just treated all of them. Some of the warts were really bad. I imagined how hard it must have been for them to access treatment out there. So I just did what I could to help.” – Clinician, man.

While it could be tempting to segregate services for YMSM within the tertiary academic institutions in a bid to let YMSM know that these services are available, HCPs felt keeping services integrated is better in terms of maintaining anonymity.

“If somebody is an MSM and you have a youth-friendly clinic where most services are integrated, it can be a solution to encourage MSM to seek services as nobody will know what they are coming for except the MSM and providers.” **–** Nurse, man.

Further, having an inclusive health climate was proposed as one of the ways of increasing uptake of services by YMSM. First, HCPs suggested having information, educational and communication materials in the health facilities that spoke to the needs of YMSM and not just those of heterosexual students.

“Do they (YMSM) feel comfortable? Do they feel welcome? For example, the posters that we put in our clinics? Are they inclusive, or just for straight people? Or do they read the message that “here I think we are not needed”. So the setup of our clinics needs to be inclusive.” – Pharmaceutical technologist, woman.

Second, HCPs suggested collecting data on sexual practices during on-campus health outreaches. By having a section in data forms that asks if the student has same-sex relations, the attending HCP would know that they are attending to YMSM. This could also make YMSM feel recognized, which could possibly encourage them to seek services at the health facilities in future, if and when they needed to.

“I think to encourage MSM to come for services, maybe during the campus outreaches we hold, we can have questionnaires then students can fill them out as they come for testing (for HIV). You can have a question that asks ‘if you are a man who has sex with men’… or something like that. So, instead of us guessing whether they are MSM, if we can include some of those questions in those questionnaires, then maybe they can come out and access the services even after the outreaches are over.” – Laboratory technologist, woman.

#### Awareness creation and advocacy for YMSM services

HCPs suggested vigorous awareness creation and advocacy efforts, akin to those at the onset of the HIV epidemic which created awareness about the epidemic at all levels of society and made several calls for action geared toward responding to the epidemic. This could increase the acceptance of MSM at the institutional level, and reassure YMSM that they are free to seek health services.

“Perhaps the approach should be like how it was with HIV. It was through years of training, political goodwill, policy changes and even being outspoken at the grassroots in mosques and churches… so that MSM know they are accepted, they are not stigmatized and they can seek services.” – Clinician, woman.

## Discussion

Our study of healthcare providers’ attitudes and perspectives toward provision of services to YMSM at tertiary academic institutions revealed three main themes, namely: healthcare providers were obligated to care for YMSM despite having non-affirming attitudes towards same-sex practices, HCPs experienced service delivery challenges when attending to, and identified opportunities for improving care for YMSM. All HCPs noted that they were duty-bound to provide services to all people (including YMSM), though the attitudes of many HCPs toward same-sex practices were subtly stigmatizing. The expression of non-affirming attitudes in our study did not seem to follow a gender or age pattern, with these attitudes being expressed by both men and women, as well as young and old HCPs. As well, there did not seem to be perceptible differences in attitudes between cadres. This lack of differences across gender, age and cadre is possibly because attitudes toward same-sex practices in Kenya are largely influenced by societal norms and religious beliefs [49]. Challenges encountered included: inadequate knowledge about the health needs of YMSM and skills to address these needs, parental versus HCP role conflict that tends to divert attention from attending to YMSM’s presenting health needs to attempts to converting YMSM to heterosexual orientation, secondary stigma from other HCPs, and unconducive healthcare settings that make it difficult for YMSM to disclose and discuss same-sex practices with HCPs. Proposals for improving care focused on: sensitizing and training of HCPs on how to provide non-stigmatizing, non-discriminatory and competent care to YMSM, encouraging more HCP interaction with YMSM, reaching out to YMSM, providing YMSM-friendly services, and advocating for YMSM services.

Our finding that HCPs perceived care for YMSM as a professional duty, is similar to a study conducted among HCPs in Malawi in 2019 [50]. Some HCPs in our study noted that while they did not accept homosexuality as a way of life, they were still able to put aside their personal beliefs and values and respond to their duty of providing care to YMSM. Besides their professional call of duty, HCPs were motivated to provide care to YMSM because “YMSM are human beings who have a right to access care like any other person”. This finding is similar to studies in other settings such as South Africa [51], though in the South African study this affirmation of the rights of YMSM was made by HCPs after a sensitivity training, in a setting where same-sex practices are not criminalized. Countries such as Kenya can therefore learn lessons from what has been done to improve care for YMSM in other African countries such as South Africa where same-sex practices are legal.

Although HCPs noted that they were willing to provide care to YMSM, some of the terms used by HCPs in reference to YMSM may suggest othering of YMSM, and possibly underlying negative attitudes toward YMSM. Examples of these terms include: “these people”, “despite who they are”, “such a person”, “having sex with other men is not right”, “the scum of the earth”, “come out clean” among others. As well, our findings from previous work suggest that YMSM largely reported experiences of stigma and discrimination when they sought services from the institution-based health facilities [38], where the HCPs in the current study were drawn from. The terms used by HCPs to refer to YMSM may be considered as examples of microaggressions. Microaggressions are a covert form of discrimination that may include brief and commonplace daily verbal indignities, whether intentional or unintentional, that communicate hostile and derogatory insults toward members of marginalized groups [52]. As observed in our previous work, YMSM were highly attuned to covert expressions of homophobic prejudice and discrimination from HCPs [38]. It’s therefore possible that HCPs who wielded power over YMSM, may have been unaware of how these microaggressions displayed their underlying non-affirming attitudes toward YMSM. This may help explain the differences between YMSM’s perceptions of how HCPs treated them, and assertions by HCPs that they treated all people equally, including YMSM. On the other hand, HCPs may have used these terms to avoid using the term “men who have sex with men” which as revealed from the discussions is socially not acceptable. The apparent opposing views of the HCPs and those of YMSM could also have been occasioned by social desirability bias, where HCPs may have masked or unmasked their attitudes toward YMSM, based on how they gauged the reactions of other HCPs during the discussions. Alternatively, perhaps the discussions offered HCPs a safe space to reflect on their attitudes and previous handling of YMSM, balance this against their professional duty of care, and hence report more seemingly supportive attitudes.

The recognition by HCPs of their professional duty to provide care to YMSM can be harnessed to improve services for YMSM, especially if the HCPs are sensitized and trained to identify and respond to the health needs of YMSM. Though some HCPs demonstrated knowledge and ability to address the health needs of YMSM (such as anal STIs), other HCPs indicated they had never “heard of anal STIs in college”. The lack of pre-service training on the health needs of non-heterosexual individuals and how to respond to these needs was reported in the current study, and has also been observed in other studies in Kenya, Uganda and Tanzania [53–55]. Evidence from Kenya and South Africa suggests that sensitizing and training reduces homo-prejudice and improves knowledge and skills of HCPs in providing care to YMSM [51, 56–58], and should hence be considered for adoption and scale-up among HCPs in tertiary academic institution-based health facilities in Kenya and other similar settings.

Religious beliefs also influenced the way HCPs handled YMSM. Rejection of same-sex practices by various religions can negatively impact healthcare for MSM [59], and indeed in the current study, most HCPs acknowledged that their religions do not accept homosexuality and this was the basis of the HCPs not accepting homosexuality too. However, most HCPs indicated capacity to compartmentalize their religious beliefs and duty to care, and in so doing stated that they were able to provide services to YMSM in a way the HCPs considered non-prejudicial. Interestingly, some HCPs noted that their agreeability to provide care for YMSM was based on religious values such as the ‘golden rule’ (treating others the way one would want to be treated) and accepting those shunned by society. Given that religion is deeply set in society [60], we suggest that this positive religious effect can be leveraged to stimulate more accommodating attitudes among HCPs that can facilitate quality and equitable care for YMSM.

Cultural factors also emerged as a challenge to provision of care. Participants reported a conflict between their dual roles as HCPs and parents, sometimes resulting in them viewing YMSM as their children who in the first place should not be having sex, and should be converted to the culturally and socially accepted heterosexual orientation. The view of young people by HCPs as not old enough to seek sexual and reproductive health services has also been found to be an age-related barrier in a study of young people at a tertiary academic institution in South Africa [61]. Even in countries where same-sex practices are legal such as the USA, one study showed that YMSM are not only told that they are too young to have sex, but are also dissuaded from having sex with men and instead encouraged to get themselves girlfriends [17]. Programs are needed to create awareness among HCPs that existing ‘therapies’ to change sexual orientation lack medical merit, and create an environment in which prejudice, stigma and discrimination thrive, thus compromising the health and well-being of sexual minorities [62], including YMSM. Secondary stigmatization of YMSM-friendly HCPs by fellow HCPs calls for more efforts in sensitizing and training geared toward achieving a critical mass of YMSM-friendly HCPs. As suggested by HCPs in this study, more encounters with YMSM clients could be helpful in fostering positive attitudes among HCPs. Considerations should be made to offer HCPs in tertiary institution-based health facilities, the opportunity to work in YMSM-friendly clinics so as to acquire hands-on experience providing services to YMSM. Indeed, evidence from a meta-analysis shows that positive contact with MSM can help reduce sexual prejudice and foster more affirming attitudes [63]. For instance, findings from a study conducted in Western Kenya demonstrated that prior HCP-MSM interaction led to reduced sexual prejudice which was in turn positively associated with comfort to provide care to MSM [64]. In addition to HCP-YMSM interactions at the clinic level, the role of intergroup dialogue or speaker series led by YMSM should be explored, though evidence from such an intervention has shown that the effect on reducing sexual prejudice is short-lived [65], and the intervention may need to be implemented over a longer period (not one-off) so as to have the desired impact.

HCPs suggested a range of approaches to address the challenge of non-disclosure of same-sex practices by YMSM. As a first step, HCPs proposed reaching out to YMSM through the orientation program for new students, and using social media channels for continuing students, in order to improve access and use of services. The World Health Organization recommends that proven youth-friendly services are adapted for YMSM, by including components pertinent for reaching and providing services to YMSM [66]. Though YMSM may have unique healthcare needs, having a specialized clinic or clinic day at the health facilities would likely be counterproductive and keep them away from seeking services. Evidence shows that in some instances, YMSM think of specialized clinics as isolating and labelling from other young people, and this may lead to avoidance of such clinics by YMSM [67]. Indeed, in a tertiary institution’s setting where students stay and learn together, having services for YMSM integrated with services for the general student population would be ideal. This way, YMSM can walk in and receive the services they need without loss of privacy or confidentiality especially regarding their sexuality and sexual health needs. Since disclosure poses the risk of stigma and discrimination [21–26], future research should explore YMSM perspectives about disclosing same-sex practices to HCPs, and how they would like to be supported if they needed to disclose. As well, adapting youth-friendly services to also being YMSM-friendly will likely require the involvement of both health facility managers and HCPs, since the HCPs cannot do this on their own without the approval and support of the managers. To address the challenge of non-acceptance of YMSM in society, HCPs recommended increased and sustained awareness creation and advocacy at various levels of the society. Though HCPs in this study were aware of the criminalization of same-sex practices under the Kenyan law, this did not seem to be a barrier to providing care to YMSM. A possible explanation for this could be the understanding by HCPs that despite the criminalization of same-sex practices in Kenya, the government through the ministry of health runs a dedicated national program that leads the HIV/STI response among key populations, including MSM [31]. Perhaps then this understanding provides an environment that can be leveraged to sensitize and train HCPs on the health needs of YMSM, and how to respond to these needs.

Our study had some limitations as well as strengths. In terms of limitations, study participants were drawn from health facilities in public tertiary institutions within the Nairobi metropolis. As a result, the views of these HCPs may not represent the views of HCPs from private tertiary institutions, as well as health facilities outside tertiary institutions, and other geographical settings in the country. However, we note that most participants had previously worked in health facilities outside the tertiary institutions and their experiences from those health facilities may have enriched the discussions. As we enlisted the support of the heads of facilities to recruit participants, it is likely that this would have introduced selection bias in our study. For instance, if the head of a facility had a supportive attitude toward same-sex practices, they would be more inclined to select participants with similar attitudes, and vice-versa. However, as noted earlier, our findings did not show perceptible distinctions across gender, age or cadre of participants, suggesting that if any, selection bias had a limited effect on the data collected. Strength wise, including HCPs of six different cadres enabled us to obtain a wider range of views, compared to other studies which mostly focus on clinicians and nurses only. This is important since all these cadres of HCPs interact with YMSM, and how YMSM are handled at various points of service from the time they enter to the time they leave the health facility, determines how well they are able to engage with healthcare services, and their likelihood to return if they needed to.

## Conclusion

All HCPs were generally cognizant of their professional duty to provide care to YMSM, but largely showed disapproval of same-sex practices, and encountered challenges in providing services to YMSM. As such, there is urgent need to implement interventions suggested by the HCPs such as training to equip them with the knowledge and skills required to address the unique health needs of YMSM. This should go along with increasing opportunities for more contact between HCPs and YMSM to help reduce homo-prejudice among HCPs, thereby fostering affirming attitudes toward YMSM. Sexual and reproductive health services offered to the student community should also be made friendly and inclusive for YMSM, to ensure no one is left behind.

### Electronic supplementary material

Below is the link to the electronic supplementary material.

Supplementary Material 1

## Data Availability

The datasets generated and analyzed during the current study are not publicly available since they are qualitative discussions with potentially identifiable information from healthcare providers discussing a topic concerning members of a criminalized and marginalized population. However, the datasets are available from the corresponding author on reasonable request.

## List of abbreviations

FGDs: Focus group discussions
HCPs: Healthcare providers
HIV: Human immuno-deficiency virus
MSM: Men who have sex with men
STIs: Sexually transmitted infections
YMSM: Young gay, bisexual and other men who have sex with men

## References

[1] Arnett JJ, Emerging Adulthood (2000). A theory of Development from the late teens through the Twenties. Am Psychol.

[2] Meyer IH (2013). Prejudice, social stress, and mental health in lesbian, gay, and bisexual populations: conceptual issues and research evidence. Psychol Sex Orientat Gend Divers.

[3] Needham BL, Austin EL, Sexual, Orientation. Parental Support, and Health During the Transition to Young Adulthood. J Youth Adolesc [Internet]. 2010;39(10):1189–98. 10.1007/s10964-010-9533-6. 10.1007/s10964-010-9533-6 20383570

[4] Toomey RB, Russell ST. The Role of Sexual Orientation in School-Based Victimization: A Meta-Analysis. Youth Soc [Internet]. 2013;48(2):176–201. 10.1177/0044118X13483778. 10.1177/0044118X13483778 PMC4795832 26997680

[5] Fields EL, Bogart LM, Smith KC, Malebranche DJ, Ellen J, Schuster MA (2015). I always Felt I had to prove my Manhood : homosexuality, masculinity, gender role strain, and HIV Risk among Young Black Men who have sex with men. Am J Public Health.

[6] Halkitis PN, Maiolatesi AJ, Krause KD (2020). The Health Challenges of Emerging Adult Gay Men: effecting change in Health Care. Pediatr Clin North Am.

[7] Koedijk FD, Van Benthem BH, Vrolings EM, Zuilhof W, Van Der Sande MA (2014). Increasing sexually transmitted infection rates in young men having sex with men in the Netherlands, 2006–2012. Emerg Themes Epidemiol.

[8] Fromme K, Corbin WR, Kruse MI (2008). Behavioral risks during the transition from High School to College. Dev Psychol.

[9] Renn K. LGBTQ Students on Campus: Issues and Opportunities for Higher Education Leaders [Internet]. Higher Education Today. 2017 [cited 2021 Jun 10]. Available from: https://www.higheredtoday.org/2017/04/10/lgbtq-students-higher-education/.

[10] Fan S, Yang Z, Hou F, Yu M, Luo Z, Liao M (2019). HIV and syphilis and sexual risk behaviours among men who have sex with men attending university in China: a systematic review and meta-analysis. Sex Health.

[11] Lucas Ramon M, Kellyn B, Rafael Carrano L, Enrique López de la P, Ilia S, Daron T. State-Sponsored Homophobia 2020: Global Legislation Overview Update [Internet]. Geneva, ILGA; 2020. Available from: https://ilga.org/downloads/ILGA_World_State_Sponsored_Homophobia_report_global_legislation_overview_update_December_2020.pdf.

[12] Santos G, Makofane K, Arreola S, Do T, Ayala G (2017). Reductions in access to HIV prevention and care services are associated with arrest and convictions in a global survey of men who have sex with men. Sex Transm Infect.

[13] Semugoma P, Nemande S, Baral SD. The irony of homophobia in Africa. Lancet [Internet]. 2012;380(9839):312–4. 10.1016/S0140-6736(12)60901-5. 10.1016/S0140-6736(12)60901-5 22819661

[14] Bhattacharjee P, Morales GJ, Kilonzo TM, Dayton RL, Musundi RT, Mbole JM (2018). Can a national government implement a violence prevention and response strategy for key populations in a criminalized setting ? A case study from Kenya. J Int AIDS Soc.

[15] Beck J, Santos G-M, Ayala G. Young Men who have sex with men: Health, Access, and HIV. The Global Forum on MSM and HIV (MSMGF); 2013.

[16] Brooks H, Llewellyn CD, Nadarzynski T, Pelloso FC (2018). Sexual orientation disclosure in health care: a systematic review. Br J Gen Pract.

[17] Griffin M, Krause KD, Kapadia F, Halkitis PN (2018). A qualitative investigation of Healthcare Engagement among Young Adult Gay Men in New York City: a P18 cohort Substudy. LGBT Heal.

[18] Logie CH, Lacombe-duncan A, Brien N, Jones N, Lee-foon N, Levermore K et al. Barriers and facilitators to HIV testing among young men who have sex with men and transgender women in Kingston, Jamaica : a qualitative study. J Int AIDS Soc [Internet]. 2017;20(1):21385. 10.7448/IAS.20.1.21385. 10.7448/IAS.20.1.21385 PMC5515029 28406274

[19] Metheny N, Stephenson R. Disclosure of Sexual Orientation and Uptake of HIV Testing and Hepatitis Vaccination for Rural Men Who Have Sex With Men. Ann Fam Med [Internet]. 2016;14(2):155 LP – 158. Available from: http://www.annfammed.org/content/14/2/155.abstract. 10.1370/afm.1907 PMC4781519 26951591

[20] Kokogho A, Amusu S, Baral SD, Charurat ME, Adebajo S, Nowak RG et al. Disclosure of Same – Sex Sexual Practices to Family and Healthcare Providers by Men Who Have Sex with Men and Transgender Women in Nigeria. Arch Sex Behav [Internet]. 2021;50(4):1665–76. 10.1007/s10508-020-01644-8. 10.1007/s10508-020-01644-8 PMC8017753 32193812

[21] Risher K, Adams D, Sithole B, Ketende S, Kennedy C, Mnisi Z (2013). Sexual stigma and discrimination as barriers to seeking appropriate healthcare among men who have sex with men in Swaziland. J Int AIDS Soc.

[22] Wanyenze RK, Musinguzi G, Matovu JKB, Kiguli J, Nuwaha F, Mujisha G (2016). If you tell people that you had sex with a fellow man, it is hard to be helped and treated : Barriers and Opportunities for increasing Access to HIV Services among Men who have sex with men in Uganda. PLoS ONE.

[23] Kushwaha S, Lalani Y, Maina G, Ogunbajo A, Wilton L, Agyarko-Poku T (2017). But the moment they find out that you are MSM ⋯: a qualitative investigation of HIV prevention experiences among men who have sex with men (MSM) in Ghana’s health care system. BMC Public Health.

[24] Rispel LC, Metcalf CA, Cloete A, Moorman J, Reddy V, Moormand J (2011). You become afraid to tell them that you are gay: health service utilization by men who have sex with men in south african cities. J Public Health Policy.

[25] Duby Z, Nkosi B, Scheibe A, Brown B, Bekker L-G. ‘Scared of going to the clinic’: Contextualising healthcare access for men who have sex with men, female sex workers and people who use drugs in two South African cities. South Afr J HIV Med [Internet]. 2018;19(1):1–8. Available from: http://sajhivmed.org.za/index.php/hivmed/article/view/701. 10.4102/sajhivmed.v19i1.701 PMC5843994 29568645

[26] Lane T, Mogale T, Struthers H, Mcintyre J, Kegeles SM (2009). They see you as a different thing: the Experiences of Men who have sex with men with Health Care Workers in South African Township Communities. Sex Transm Infect.

[27] Beyrer C, Sullivan PS, Sanchez J, Dowdy D, Altman D, Trapence G et al. A call to action for comprehensive HIV services for men who have sex with men. Lancet [Internet]. 2012;380(9839):424–8. 10.1016/S0140-6736(12)61022-8. 10.1016/S0140-6736(12)61022-8 PMC3805059 22819663

[28] Elst EM, Van Der, Kombo B, Gichuru E, Omar A, Musyoki H, Graham SM (2015). The green shoots of a novel training programme: progress and identified key actions to providing services to MSM at kenyan health facilities. J Int AIDS Soc.

[29] The Government of Kenya. Laws of Kenya Cap 63: The Penal Code [Internet]. Kenya: Kenya Law. ; 1930. Available from: http://www.kenyalaw.org/lex/actview.xql?actid=CAP. 63.

[30] Wanga S. Supreme Court: Gays and lesbians have a right of association. The Standard [Internet]. 2023; Available from: https://www.standardmedia.co.ke/national/article/2001467772/supreme-court-gays-and-lesbians-have-a-right-of-association.

[31] National AIDS, and STI Control Programme (NASCOP) Kenya. National AIDSSTI Control Programme Organogram [Internet]. [cited 2022 Jul 9]. Available from: https://www.nascop.or.ke/organogram/.

[32] Daily Active. Nairobi’s very own gay clinic with a surprising number of patients. Daily Active [Internet]. 2019; Available from: https://dailyactive.info/2019/05/24/a-look-at-nairobis-very-own-gay-clinic-with-a-surprising-number-of-same-sex-patients/.

[33] Bourne A, Carman M, Kabuti R, Nutland W, Fearon E, Liku J et al. Experiences and challenges in sexual health service access among men who have sex with men in Kenya. Glob Public Health [Internet]. 2022;17(8):1626–37. 10.1080/17441692.2021.1987501. 10.1080/17441692.2021.1987501 34632949

[34] Mwaniki SW, Mugo PM, Palanee-Phillips T, Project BESPOKE. (Integrated Bio-Behavioral Assessment of HIV and STI Among Young Tertiary Student Men Who Have Sex With Men in Nairobi, Kenya): A Respondent-Driven Sampling Survey Protocol. Front Public Heal [Internet]. 2021;9:619694. Available from: https://www.frontiersin.org/article/10.3389/fpubh.2021.619694. 10.3389/fpubh.2021.619694 PMC8542710 34708012

[35] Mwaniki SW, Kaberia PM, Mugo PM, Palanee-Phillips T. “My Friends Would Believe My Word”: Appropriateness and Acceptability of Respondent-Driven Sampling in Recruiting Young Tertiary Student Men Who Have Sex with Men for HIV/STI Research in Nairobi, Kenya. Int J Environ Res Public Health [Internet]. 2022;19(12):7331. Available from: https://www.mdpi.com/1660-4601/19/12/7331. 10.3390/ijerph19127331 PMC9223518 35742579

[36] Mwaniki SW, Kaberia PM, Mugo PM, Palanee-Phillips T. HIV prevalence and associated risk factors among young tertiary student men who have sex with men (MSM) in Nairobi, Kenya: a respondent-driven sampling survey. AIDS Res Ther [Internet]. 2023;20:7. 10.1186/s12981-023-00502-6. 10.1186/s12981-023-00502-6 PMC9900555 36747178

[37] Mwaniki SW, Kaberia PM, Mugo PM, Palanee-Phillips T (2023). Prevalence of five curable sexually transmitted infections and associated risk factors among tertiary student men who have sex with men in Nairobi, Kenya: a respondent-driven sampling survey. Sex Health.

[38] Mwaniki SW, Kaberia PM, Mugo PM, Palanee-Phillips T. “What if I get sick, where shall I go?”: a qualitative investigation of healthcare engagement among young gay, bisexual and other men who have sex with men in Nairobi, Kenya. Res Sq [Internet]. 2022;v1. Available from: https://www.researchsquare.com/article/rs-1994768/v1. 10.1186/s12889-023-17555-x PMC10763282 38166989

[39] Frith H (2000). Focusing on sex: using Focus Groups in Sex Research. Sexualities.

[40] Morgan DL, Krueger RA. When to Use Focus Groups and Why. In: Morgan DL, editor. Successful Focus Groups: Advancing the State of the Art [Internet]. Thousand Oaks: SAGE Publications, Inc.; 2013. p. 3–19. Available from: https://methods.sagepub.com/base/download/BookChapter/successful-focus-groups/n1.xml.

[41] Study in Kenya. Universities and Colleges in Nairobi County [Internet]. 2021 [cited 2022 Jun 24]. Available from: https://studyinkenya.co.ke/universities-and-colleges-in-nairobi-county?page=1.

[42] University of Nairobi. University of Nairobi Health Services [Internet]. [cited 2022 Apr 29]. Available from: http://healthservices.uonbi.ac.ke/.

[43] Kenyatta University. Kenyatta University Health Unit [Internet]. [cited 2022 Apr 29]. Available from: http://www.ku.ac.ke/healthunit/.

[44] Jomo Kenyatta University of Agriculture and Technology. Jomo Kenyatta University of Agriculture and Technology Health Services Department [Internet]. [cited 2022 Apr 29]. Available from: http://www.jkuat.ac.ke/departments/hospital/.

[45] Guest G, Namey E, McKenna K (2017). How many focus groups are Enough? Building an evidence base for nonprobability sample sizes. Field Methods.

[46] Liamputtong P (2020). Qualitative research methods.

[47] Tong A, Sainsbury P, Craig J. Consolidated criteria for reporting qualitative research (COREQ): a 32-item checklist for interviews and focus groups. Int J Qual Heal Care [Internet]. 2007;19(6):349–57. 10.1093/intqhc/mzm042. 10.1093/intqhc/mzm042 17872937

[48] Shenton AK (2004). Strategies for ensuring trustworthiness in qualitative research projects. Educ Inf.

[49] Mucherah W, Owino E, McCoy K (2016). Grappling with the issue of homosexuality: perceptions, attitudes, and beliefs among high school students in Kenya. Psychol Res Behav Manag.

[50] Kapanda L, Jumbe V, Izugbara C, Muula AS (2019). Healthcare providers’ attitudes towards care for men who have sex with men (MSM) in Malawi. BMC Health Serv Res.

[51] Duby Z, Fong-Jaen F, Nkosi B, Brown B, Scheibe A (2019). We must treat them like all the other people’: evaluating the Integrated Key populations sensitivity Training Programme for Healthcare Workers in South Africa. South Afr J HIV Med.

[52] Nadal KL, Wong Y, Issa MA, Meterko V, Leon J, Wideman M (2011). Sexual orientation microaggressions: processes and coping mechanisms for lesbian, gay, and bisexual individuals. J LGBT Issues Couns.

[53] Taegtmeyer M, Davies A, Mwangome M, Elst EM, Van Der, Graham SM, Price MA (2013). Challenges in providing counselling to MSM in highly stigmatized Contexts: results of a qualitative study from Kenya. PLoS ONE.

[54] Matovu JKB, Musinguzi G, Kiguli J, Nuwaha F, Mujisha G, Musinguzi J (2019). Health providers’ experiences, perceptions and readiness to provide HIV services to men who have sex with men and female sex workers in Uganda - A qualitative study. BMC Infect Dis.

[55] Mgopa LR, Rosser BRS, Ross MW, Lukumay GG, Mohammed I, Massae AF (2021). Cultural and clinical challenges in sexual health care provision to men who have sex with men in Tanzania: a qualitative study of health professionals’ experiences and health students’ perspectives. BMC Public Health.

[56] Elst EM, Van Der, Gichuru E, Omar A, Kanungi J, Duby Z, Midoun M (2013). Experiences of kenyan healthcare workers providing services to men who have sex with men: qualitative findings from a sensitivity training programme. J Int AIDS Soc.

[57] Elst EM, Van Der, Smith AD, Gichuru E, Wahome E, Musyoki H, Muraguri N (2013). Men who have sex with men sensitivity training reduces homoprejudice and increases knowledge among kenyan healthcare providers in coastal Kenya. J Int AIDS Soc.

[58] Scheibe AP, Duby Z, Brown B, Sanders EJ, Bekker L-G. Attitude shifts and knowledge gains: Evaluating men who have sex with men sensitisation training for healthcare workers in the Western Cape, South Africa. South Afr J HIV Med [Internet]. 2017;18(1):a673. Available from: http://sajhivmed.org.za/index.php/hivmed/article/view/673. 10.4102/sajhivmed.v18i1.673 PMC5843261 29568621

[59] Mbote DK, Sandfort TGM, Waweru E, Zapfel A. Kenyan Religious Leaders’ Views on Same-Sex Sexuality and Gender Nonconformity: Religious Freedom versus Constitutional Rights. J Sex Res [Internet]. 2018;55(4–5):630–41. 10.1080/00224499.2016.1255702. 10.1080/00224499.2016.1255702 PMC5474220 27982708

[60] Gichuru E, Kombo B, Mumba N, Sariola S, Sanders EJ, van der Elst EM. Engaging religious leaders to support HIV prevention and care for gays, bisexual men, and other men who have sex with men in coastal Kenya. Crit Public Health [Internet]. 2018;28(3):294–305. 10.1080/09581596.2018.1447647. 10.1080/09581596.2018.1447647 PMC5935049 29770367

[61] Alli F, Maharaj P, Vawda MY (2013). Interpersonal relations between health care workers and young clients: barriers to accessing sexual and reproductive health care. J Community Health.

[62] Pan American Health Organization/World Health Organization (2012). Therapies to change sexual orientation lack medical justification and threaten health.

[63] Smith SJ, Axelton AM, Saucier DA (2009). The effects of contact on sexual prejudice: a meta-analysis. Sex Roles.

[64] Shangani S, Genberg B, Harrison A, Pellowski J, Wachira J, Naanyu V et al. Sexual Prejudice and Comfort to Provide Services to Men Who Have Sex with Men Among HIV Healthcare Workers in Western Kenya: Role of Interpersonal Contact. AIDS Behav [Internet]. 2022;26(3):805–13. 10.1007/s10461-021-03440-4. 10.1007/s10461-021-03440-4 34406550

[65] Cramwinckel FM, Scheepers DT, Wilderjans TF, de Rooij RJB. Assessing the Effects of a Real-Life Contact Intervention on Prejudice Toward LGBT People. Arch Sex Behav [Internet]. 2021;50(7):3035–51. 10.1007/s10508-021-02046-0. 10.1007/s10508-021-02046-0 PMC8563548 34505215

[66] World Health Organization (WHO) (2015). HIV and young men who have sex with men: technical brief.

[67] Laiti M, Pakarinen A, Parisod H, Salanterä S, Sariola S (2019). Encountering sexual and gender minority youth in healthcare: an integrative review. Prim Heal Care Res Dev.

