# Supplementary material for: “We must help them despite who they are…”: healthcare providers’ attitudes and perspectives on care for young gay, bisexual and other men who have sex with men in Nairobi, Kenya
Source: BMC Health Serv Res. 2023 Oct 3;23:1055. doi: 10.1186/s12913-023-10026-4 (PMC10546658; doi:10.1186/s12913-023-10026-4)
Supplement: Supplementary file 1 — Supplementary Material 1 [file 12913_2023_10026_MOESM1_ESM.docx]

**Supplementary file 1**

**Focus group discussion guide to assess healthcare providers’ attitudes and perspectives on care for young gay, bisexual and other men who have sex with men**

**Introduction:** Thank you again for your willingness to be a part of this discussion. We are looking forward to hearing your thoughts on the questions we will ask you. Please know that there are no right or wrong answers and we welcome every opinion about the topics we will discuss, so feel free to share your thoughts openly. If during our discussion, there are issues or concerns that you would like to talk about, feel free to bring them up, even if we do not ask about them.

| **Theme** | **Questions** | **Probes/ prompts** | **Notes** |
| --- | --- | --- | --- |
| Attitudes | How would you describe general attitudes towards men who have sex with men (MSM) in the community where you live? | - What makes you say this? - What do people say about MSM? - How are MSM treated by people in the community? - How would you describe the experiences of MSM living in this community? |  |
|  | Do you think there are MSM students in the institutions where you work? | - Why do you say so? |  |
|  | How would you describe general attitudes towards MSM students in the institution where you work? | - What makes you say this? - What do other students say about MSM students? - What do staff say about MSM students? - What do healthcare workers say about MSM? - How are MSM treated by people (other students/staff/healthcare providers) in the institution? - How would you describe the experiences of MSM students in learning institutions? |  |
|  | How would you feel if you came to know a student seeking health services from you is MSM? | - What would make you feel this way? - Would you judge/criticize or support them? - Would you ask them to stop having sex with men? - Would you ask them to change? |  |
|  | Would you say you have any feelings/beliefs/attitudes/opinions that would make it difficult for you to provide services to MSM students? | - What are these feelings/beliefs/attitudes/opinions? - Do you think anything can be done to change these feelings/beliefs/attitudes/opinions? |  |
| Perspectives | Would you say that MSM students are able to access sexual health services from the health facilities in the learning institutions? | - How easy or difficult do you think it is for MSM students to access sexual health services? - How important do you think it is for MSM students to access sexual health services in the institutions? - What do you think is the best way to encourage MSM students to come for sexual health services in the institutions? |  |
|  | Have you had experience providing services to MSM students? | - What services did you provide? - How was the experience? - Have you had any particular challenges serving MSM students? - How about successes? |  |
|  | Supposing there was a training on providing services to MSM students, what would be your thoughts about such a training? | - Would you be willing/not willing to participate? - Why would you be willing/not willing to participate? - How useful would that training be for you? - Would it improve your knowledge and attitudes towards MSM students? |  |

**Conclusion:** Thank you everyone for everything you have shared with us. We have learnt so much. Thank you once again and enjoy the rest of your day.
